# Janelas Pericárdicas: O Valor Limitado do Diagnóstico da Biópsia Pericárdica não Guiada

**DOI:** 10.36660/abc.20230082

**Published:** 2023-09-28

**Authors:** Gabriel Bianco Giuliani, Ismael Alberto Auca Morales, Giovane Okaresnki, Gabriel Faioli Nascimento Alves Vieira, Daniella de Freitas Pereira Angelo Durço, Alfredo José Rodrigues

**Affiliations:** 1 Departamento de Cirurgia e Anatomia Faculdade de Medicina de Ribeirão Preto Universidade de São Paulo Ribeirão Preto SP Brasil Departamento de Cirurgia e Anatomia da Faculdade de Medicina de Ribeirão Preto da Universidade de São Paulo, Ribeirão Preto, SP – Brasil; 2 Departamento de Patologia Faculdade de Medicina de Ribeirão Preto Universidade de São Paulo Ribeirão Preto SP Brasil Departamento de Patologia da Faculdade de Medicina de Ribeirão Preto da Universidade de São Paulo, Ribeirão Preto, SP – Brasil

**Keywords:** Pericardio/ultraestrutura, Biópsia não guiada, Derrame Pericárdico, Tamponamento Cardíaco

## Abstract

**Fundamento:**

A janela pericárdica, além de promover a drenagem pericárdica, também pode fornecer amostras do pericárdio para exame anatomopatológico. No entanto, a contribuição dessas biópsias para a elucidação da etiologia do derrame pericárdico tem sido debatida.

**Objetivo:**

Analisar o valor diagnóstico da biópsia pericárdica não guiada obtida de procedimentos de janela pericárdica.

**Métodos:**

Foram revisados retrospectivamente dados de 80 pacientes submetidos a biópsia pericárdica parietal de 2011 a 2020. A significância estatística foi considerada quando p < 0,05.

**Resultados:**

Cinquenta pacientes eram do sexo masculino (62,5%) e 30 do sexo feminino (37,5%). A mediana de idade foi de 52 anos (intervalo interquartil: 29 a 59) e 49 anos (intervalo interquartil: 38 a 65), respectivamente (p = 0,724). A etiologia suspeita do derrame pericárdico foi neoplásica em 31,3%, incerta em 25%, tuberculose em 15%, autoimune em 12,5%, síndrome edemigênica em 7,5% e outras condições diversas em 8,8%. A abordagem mais frequente para drenagem pericárdica e biópsia foi a subxifoide (74%), seguida pela videotoracoscopia (22%). Em 78,8% das biópsias, os achados histopatológicos foram compatíveis com inflamação inespecífica, e apenas 13,7% de todas as biópsias produziram um diagnóstico histopatológico conclusivo. Aqueles que sofriam de câncer e derrame pericárdico apresentaram maior proporção de achados histopatológicos conclusivos (32% apresentavam infiltração neoplásica pericárdica). A taxa de mortalidade hospitalar foi de 27,5% e 54,5% dos pacientes que morreram no hospital tinham câncer. Nenhuma morte foi atribuída ao tamponamento cardíaco ou ao procedimento de drenagem.

**Conclusão:**

Nossos resultados mostraram que a janela pericárdica é um procedimento seguro, mas teve pouco valor para esclarecer a etiologia do derrame pericárdico e nenhum impacto na terapia planejada para o diagnóstico primário além da descompressão cardíaca.

**Figura Central:**
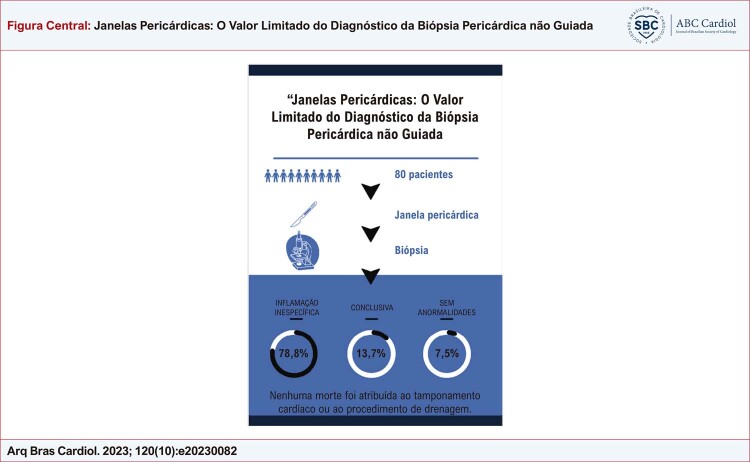
: Janelas Pericárdicas: O Valor Limitado do Diagnóstico da Biópsia Pericárdica não Guiada

## Introdução

O derrame pericárdico não é uma condição incomum. Pode ser assintomático ou apresentar sinais clínicos de comprometimento funcional cardiovascular devido ao tamponamento cardíaco. A etiologia do derrame pericárdico varia de acordo com características demográficas e comorbidades existentes. As causas mais citadas são infecções, cirurgia cardíaca, inflamatórias/reumatológicas, neoplásicas e idiopáticas,^1^ e muitas estão associadas a condições médicas conhecidas, como insuficiência renal crônica e outras síndromes edemigênicas.

A pericardiocentese subxifoide, descrita pela primeira vez por Marfan,^2^ é um procedimento minimamente invasivo que pode ser realizado com anestesia local à “beira do leito.” Além de descomprimir o coração, fornece uma amostra de fluido para fins de diagnóstico. No entanto, esse método geralmente não fornece amostras de tecido pericárdico para exame anatomopatológico.

A janela pericárdica, ou fenestração, é um procedimento realizado por meio de diversas abordagens cirúrgicas abertas (subcostal, esternotomia, toracotomia, toracoscópica, subxifoide), que, além de promover a drenagem pericárdica, também pode fornecer amostras do pericárdio para exame anatomopatológico. A abordagem subxifoide, conhecida como “janela subxifoide”, é comumente utilizada, pois não requer instrumentos e equipamentos especiais e preserva o espaço pleural e o esterno, podendo ser realizada com anestesia local.

Apesar da eficácia da janela pericárdica para resolver o tamponamento cardíaco, a contribuição das amostras pericárdicas não guiadas de padrão, obtidas usando tais abordagens para esclarecer a etiologia do derrame, é questionável.^3 , 4^

Portanto, o objetivo do presente estudo foi analisar o valor diagnóstico da biópsia pericárdica não guiada obtida de procedimentos de janela pericárdica.

## Métodos

Foram revisadas todas as amostras do pericárdio parietal excisadas cirurgicamente no Hospital das Clínicas da Faculdade de Medicina de Ribeirão Preto da Universidade de São Paulo, Brasil, de 2011 a 2020. A história e evolução clínica, bem como a ecocardiografia e os resultados histológicos foram obtidos retrospectivamente através do acesso aos prontuários médicos dos pacientes.

Todas as biópsias foram obtidas durante janela pericárdica realizada por abordagem subxifoide, videotoracoscopia ou toracotomia sob anestesia geral. O presente estudo foi aprovado pelo comitê de ética em pesquisa do Hospital das Clínicas da Faculdade de Medicina de Ribeirão Preto da Universidade de São Paulo (CAAE: 65868422.3.0000.5440).

### Análise estatística

A distribuição dos dados foi avaliada usando histogramas e gráficos Q-Q. Como as variáveis contínuas não apresentaram distribuição normal, seus resultados são apresentados como medianas e primeiro e terceiro quartis (Q1 a Q3). Os resultados para variáveis categóricas são apresentados como proporções. As variáveis contínuas e categóricas foram comparadas pelo teste de Mann-Whitney e o teste exato de Fisher, respectivamente. A significância estatística foi considerada quando p < 0,05. A análise foi realizada no programa SPSS for Windows (IBM® SPSS®), versão 25.

## Resultados

Foram revisados dados de 80 pacientes. Cinquenta pacientes eram do sexo masculino (62,5%) e 30 do sexo feminino (37,5%). A mediana de idade foi de 52 anos (intervalo interquartil: 29 a 59) e 49 anos (intervalo interquartil: 38 a 65), respectivamente (p = 0,724). De acordo com os parâmetros ecocardiográficos, o derrame pericárdico foi considerado pequeno em 17% dos pacientes, moderado em 28% e grande em 55%. O derrame pleural concomitante esteve presente em 59% dos pacientes, independentemente do volume do derrame pericárdico (p = 0,394). Apenas 5% de todos os pacientes necessitaram de drenagem pericárdica urgente.

A etiologia suspeita do derrame pericárdico, baseada apenas na história clínica e exames diagnósticos não invasivos, foi neoplásica em 31,3%, incerta em 25%, tuberculose em 15%, autoimune em 12,5%, síndrome edemigênica (cirrose, insuficiência cardíaca congestiva, síndrome de consumo, insuficiência renal) em 7,5% e outras condições diversas em 8,8%.

No geral, 11 pacientes (13,8%) tiveram drenagem pericárdica prévia, dos quais 63,6% foram por pericardiocentese subxifoide e os demais por janela subxifoide. Entre os pacientes com drenagem pericárdica prévia, 45,5% tinham câncer; 18,2% apresentavam síndrome edemigênica e o diagnóstico clínico não era claro em 18,2%. A Tabela 1 mostra as características clínicas e sintomas de todos os pacientes.

**Tabela 1:** – Características clínicas e sintomas dos pacientes

Fração de ejeção do ventrículo esquerdo		0,65 (0,57-0,69)
Hemoglobina (g/dl)		10,9 (9-13)
Hematócrito (%)		32,5 (28-39)
Plaquetas (× 10 ^3^ /µl)		286 (193-380)
Leucócitos (× 10 ^3^ /mm ^3^ )		8 (5-12)
Proteína C reativa (mg/L)		7,3 (2-17)
Distensão venosa jugular		22,7%
Sons cardíacos abafados		16,7%
Pulso paradoxal		1,5%
Pressão arterial sistólica < 100 mmHg		10,8%
Tríade de Beck		0,0%
Dispneia		43,9%
Ortopneia		13,6%
Dispneia paroxística noturna		6,1%
Classe NYHA	I	62,7%
	II	23,7%
	III	8,5%
	IV	5,1%
Febre		10,6%
Dor torácica		13,6%
Ascites		7,5%
Fricção pericárdica		1,5%
Fadiga		28,8%
Derrame pleural		65,3%
Derrame pericárdico prévio		15,5%
Hipotireoidismo		16,7%
Câncer		33,8%
Insuficiência renal		22,5%

As variáveis contínuas são apresentadas como medianas e quartis (Q1 a Q3).

A abordagem mais frequente para drenagem e biópsia pericárdica foi a subxifoide (74%), seguida de videotoracoscopia (22%) e toracotomia (4%). Em relação aos achados ecocardiográficos associados ao tamponamento cardíaco,^5^ no geral, 34% apresentaram colapso sistólico do átrio direito; 24% apresentaram colapso diastólico do ventrículo direito; 46% apresentaram uma diminuição de 25% ou mais na velocidade de influxo da válvula mitral durante a inspiração; 41% apresentaram aumento de pelo menos 40% na velocidade de influxo da valva tricúspide durante a inspiração; e 43% não apresentaram colapso de nenhuma câmara (átrio direito ou ventrículo direito). Os sintomas, sinais e achados ecocardiográficos de acordo com o volume de derrame determinado pelo ecocardiograma transtorácico são apresentados na Tabela 2 .

**Tabela 2:** – Sintomas, sinais e achados ecocardiográficos de acordo com o volume de derrame determinado pelo ecocardiograma transtorácico

Sintomas e sinais	Volume							p

	Pequeno			Moderado		Grande		
		
		n	%	n	%	n	%	
Distensão venosa jugular		2	20,0%	2	11,1%	11	31,4%	0,252
Sons cardíacos abafados		1	10,0%	2	11,1%	8	22,9%	0,586
Pulso paradoxal		1	10,0%	0	0,0%	0	0,0%	0,159
Pressão arterial sistólica < 100mmHg		0	0,0%	2	11,1%	5	14,7%	0,655
Tríade de Beck		0	0,0%	0	0,0%	0	0,0%	-
Dispneia		5	50,0%	6	33,3%	18	51,4%	0,418
Ortopneia		0	0,0%	4	22,2%	5	14,3%	0,334
Dispneia paroxística noturna		1	10,0%	2	11,1%	1	2,9%	0,360
Esforço		6	60,0%	5	27,8%	8	22,9%	0,091
Classe NYHA	I	6	60,0%	12	70,6%	17	56,7%	0,907
	II	3	30,0%	4	23,5%	7	23,3%	
	III	1	10,0%	1	5,9%	3	10,0%	
	IV	0	0,0%	0	0,0%	3	10,0%	
Colapso sistólico do AD		0	0,0%	7	35,0%	17	44,7%	0,029* 0,004†
Colapso diastólico do VD		1	8,3%	4	20,0%	12	31,6%	0,284
Diminuição de 25% na velocidade de influxo da VM durante inspiração		2	16,7%	8	40,0%	22	57,9%	0,019†
Aumento de 40% na velocidade de influxo da VT durante inspiração		2	16,7%	4	20,0%	23	60,5%	0,018† 0,005‡

*valor de p para pequeno versus moderado; † valor de p para pequeno versus grande; ‡ valor de p para moderado versus grande; AD: átrio direito; VD: ventrículo direito; VM: valva mitral; VT: válvula tricúspide.

Apenas 13,7% de todas as biópsias pericárdicas produziram um diagnóstico histopatológico conclusivo. A Tabela 3 mostra os achados histopatológicos predominantes para cada etiologia suspeita de derrame pericárdico de acordo com a história clínica. No geral, em 78,8% das biópsias, os achados histopatológicos foram compatíveis com inflamação pericárdica inespecífica. De todos os pacientes com derrame pericárdico e câncer, 34,6% apresentaram infiltração pericárdica por células neoplásicas; 57,7% apresentaram achados inflamatórios inespecíficos e nenhuma anormalidade foi encontrada em 7,7%. As neoplasias mais frequentes associadas ao derrame pericárdico foram malignidades hematológicas em 30,8%, câncer de pulmão em 26,9% e câncer de mama e câncer cervical em 11,5% cada. A maior proporção de biópsias com envolvimento pericárdico neoplásico foram aquelas realizadas em pacientes com câncer de pulmão ou de mama (positividade de 71,4% e 66,7%, respectivamente).

**Tabela 3:** – Achados histopatológicos predominantes para cada etiologia suspeita de derrame pericárdico de acordo com a história clínica

Etiologias suspeitas	Achado histopatológico									

	Sem anormalidades		Achados inflamatórios inespecíficos				Amiloidose		Infiltração neoplásica	

			Crônica		Aguda					
				
	n	%	n	%	n	%	n	%	n	%
Diagnóstico incerto	2	10,0%	12	60,0%	5	25,0%	0	0,0%	1	5,0%
Neoplásica	2	8,0%	15	60,0%	0	0,0%	0	0,0%	8	32,0%
Doença autoimune	2	20,0%	7	70,0%	1	10,0%	0	0,0%	0	0,0%
Tuberculose	0	0,0%	10	83,3%	2	16,7%	0	0,0%	0	0,0%
Outras	0	0,0%	6	85,7%	0	0,0%	1	14,3%	0	0
Síndrome edemigênica	0	0,0%	3	50,0%	2	33,3%	1	16,7%	0	0,0%

A taxa de mortalidade hospitalar geral foi de 27,5%, com mediana de idade de 52 anos (44 a 67), e 68,2% eram do sexo feminino. Nenhuma morte foi atribuída ao tamponamento cardíaco ou ao procedimento de drenagem, e 54,5% dos que foram a óbito no hospital tiveram câncer. Os demais óbitos ocorreram em pacientes com múltiplas comorbidades crônicas. Além do diagnóstico de câncer (mortalidade de 50% versus 16,7%, p = 0,003), nenhuma outra variável clínica ou ecocardiográfica esteve associada ao óbito hospitalar ( Tabela 4 ).

**Tabela 4:** – Sintomas, sinais e achados ecocardiográficos de acordo com o desfecho

		Sobrevida		Óbito hospitalar		p

		n		n		
Idade (anos)		58	49 (35-59)	22	52 (44-67)	0,380
Sexo feminino		35	60,3%	15	68,2%	0,610
Colapso sistólico do AD		17	33,3%	7	36,8%	0,784
Colapso diastólico do VD		11	21,6%	6	31,6%	0,531
Diminuição de 25% na velocidade de influxo da VM		0	0,0%	0	0,0%	-
Aumento de 40% na velocidade de influxo da VT		22	43,1%	10	52,6%	0,592
Colapso sistólico do AD		19	37,3%	10	52,6%	0,283
Volume do derrame	Pequeno	8	15,4%	4	21,1%	0,872
	Moderado	15	28,8%	5	26,3%	
	Grande	29	55,8%	10	52,6%	
Fração de ejeção do ventrículo esquerdo		0,65 (60-69)		0,62 (0,55-0,70)		0,466
Distensão venosa jugular		10	20,4%	5	29,4%	0,508
Sons cardíacos abafados		8	16,3%	3	17,6%	0,900
Pulso paradoxal		1	2,0%	0	0,0%	1,0
Pressão arterial sistólica < 100 mmHg		5	10,4%	2	11,8%	1,0
Tríade de Beck		19	38,8%	10	58,8%	0,169
Dispneia		5	10,2%	4	23,5%	0,220
Ortopneia		2	4,1%	2	11,8%	0,271
Classe NYHA	I	29	64,4%	8	57,1%	0,854
	II	10	22,2%	4	28,6%	
	III	4	8,9%	1	7,1%	
	IV	2	4,4%	1	7,1%	

As variáveis contínuas são apresentadas como medianas e quartis (Q1 a Q3). AD: átrio direito; VD: ventrículo direito; VM: valva mitral; VT: válvula tricúspide.

## Discussão

Nossos resultados mostraram que a janela pericárdica é um procedimento seguro, mas amostras pericárdicas não guiadas obtidas da janela pericárdica realizada tiveram pouco valor na elucidação da etiologia do derrame pericárdico e nenhum impacto na terapia planejada para o diagnóstico primário além da descompressão cardíaca. Nossos resultados também mostraram que 43% dos pacientes não apresentavam sinais ecocardiográficos de qualquer colapso de câmara (átrio ou ventrículo direito), um achado com valor preditivo negativo de 90% para tamponamento.^5^

A utilidade de biópsias pericárdicas não guiadas obtidas como parte de procedimentos terapêuticos, como janelas pericárdicas, tem sido questionada. Fernandes et al.,^6^ verificaram que a biópsia pericárdica revelou a etiologia do derrame pericárdico em apenas 10,5% de 38 pacientes. Na experiência de Boldes et al.,^3^ fibrose foi encontrada em 71% de todas as amostras coletadas; achados inflamatórios estiveram presentes em 86,2%, e o valor diagnóstico da biópsia pericárdica em neoplasias metastáticas do pericárdio teve sensibilidade global de 57,69%. Os autores concluíram que essas biópsias não alteraram o diagnóstico primário. Volk et al.,^4^ também observaram que o diagnóstico foi possível em apenas 23,9% dos casos com a drenagem cirúrgica do derrame pericárdico realizada por via subxifoide ou toracotomia.

**Figura 1:**
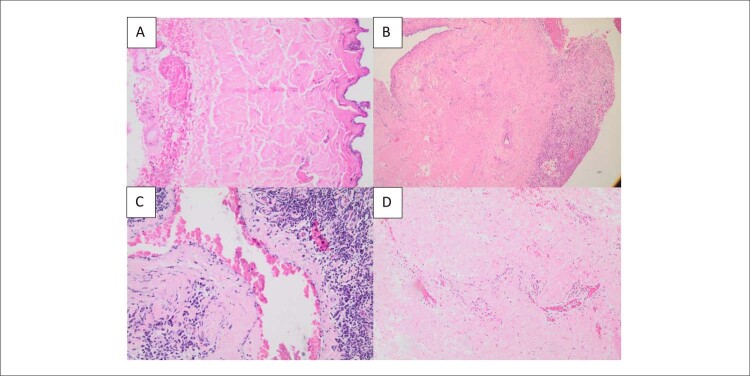
– Achados histopatológicos. A) Normal: Camada parietal de pericárdio seroso, pericárdio fibroso e tecido adiposo adjacente sem alterações histopatológicas; B) Neoplasia: Pericárdio fibroso infiltrado por metástase de osteossarcoma de alto grau; C) Inflamação crônica: Pericárdio fibroso exibindo denso infiltrado inflamatório crônico linfoplasmocitário perivascular; D) Inflamação aguda: Pericárdio exibindo infiltrado inflamatório neutrofílico predominantemente perivascular com extravasamento de hemácias.

A drenagem cirúrgica do derrame pericárdico e a biópsia pericárdica realizada por via subxifoide geralmente permitem o acesso a uma porção limitada do pericárdio, diminuindo a chance de obter amostras representativas. Contudo, a pericardioscopia parece melhorar o valor diagnóstico das biópsias pericárdicas, pois permite uma inspeção mais ampla da cavidade pericárdica, com visualização de áreas suspeitas, possibilitando, consequentemente, a obtenção de múltiplas amostras guiadas.^7 , 8^

Além da contribuição da biópsia pericárdica no momento da janela pericárdica para o esclarecimento da etiologia, também merece análise mais aprofundada a questão da estratégia ideal para a drenagem dos derrames pericárdicos, se seria a pericardiocentese ou a drenagem cirúrgica.

Horr et al.,^9^ compararam os resultados de pacientes submetidos à pericardiocentese ou à janela pericárdica e concluíram que ambos os procedimentos são estratégias seguras e eficazes para pacientes com derrame pericárdico. Eles também verificaram que o reacúmulo do derrame estava associado à ausência de um dreno no local após o procedimento.^10^ No entanto, vale lembrar que a drenagem percutânea por cateter pericárdico é viável e segura, principalmente se guiada por eco, e que as características clínicas do paciente certamente têm grande influência na taxa de recorrência do derrame. Além disso, um tempo prolongado de drenagem do cateter pericárdico tem sido associado a uma recorrência reduzida de tamponamento pericárdico após pericardiocentese.^11^

Pan et al.,^12^ utilizando uma amostra nacionalmente representativa de 44.637 registros, compararam os resultados entre ambas as abordagens de drenagem, pericardiocentese ou drenagem cirúrgica aberta, em pacientes com derrame pericárdico não relacionado a cirurgia. Observaram, após ajuste de risco, que a pericardiocentese estava associada a maiores chances de mortalidade, complicações cardíacas, reintervenção e readmissão em 30 dias para drenagem cirúrgica. A pericardiocentese foi associada a menores chances de complicações infecciosas, respiratórias e hemorrágicas, mas maiores chances de complicações cardíacas, em comparação com a abordagem cirúrgica aberta.

Portanto, o debate sobre a melhor abordagem para o diagnóstico e manejo do derrame pericárdico merece mais estudos.

O presente estudo tem várias limitações importantes. Esta é uma análise retrospectiva dos prontuários dos pacientes; portanto, está sujeito aos vieses desse tipo de estudo. Adicionalmente, nossa coorte é pequena; analisamos os dados apenas durante o período de internação, e a escolha da estratégia de abordagem do derrame pericárdico foi influenciada pela experiência do operador, pelos recursos hospitalares disponíveis e pela gravidade do quadro do paciente, resultando em potencial viés.

Dito isso, nosso estudo fornece dados confiáveis sobre os achados histopatológicos de biópsias pericárdicas obtidas através de abordagens cirúrgicas de rotina, principalmente a subxifoide, que apoiam a nossa conclusão e podem ser úteis para o desenvolvimento de estratégias para tratar doenças pericárdicas.

## Conclusão

Nossos resultados mostraram que a biópsia pericárdica não guiada realizada no momento da janela pericárdica é segura, mas teve pouco valor para diagnosticar a etiologia do derrame pericárdico.
