# Supplementary material for: Janelas Pericárdicas: O Valor Limitado do Diagnóstico da Biópsia Pericárdica não Guiada
Source: Arq Bras Cardiol. 2023 Sep 28;120(10):e20230082. [Article in Portuguese] doi: 10.36660/abc.20230082 (PMC10547430; doi:10.36660/abc.20230082)
Supplement: Translation

# Translation: Pericardial Windows: The Limited Diagnostic Value of Non-Targeted Pericardial Biopsy

**Authors:** Gabriel Bianco Giuliani, Ismael Alberto Auca Morales, Giovane Okaresnki, Gabriel Faioli Nascimento Alves Vieira, Daniella de Freitas Pereira Angelo Durço, Alfredo José Rodrigues

**Affiliations:** 1 Brazil Departamento de Cirurgia e Anatomia da Faculdade de Medicina de Ribeirão Preto da Universidade de São Paulo, Ribeirão Preto, SP – Brazil; 2 Brazil Departamento de Patologia da Faculdade de Medicina de Ribeirão Preto da Universidade de São Paulo, Ribeirão Preto, SP – Brazil

**Keywords:** Pericardium/ultrastructure, Biopsy/image-non-guide, Pericardial Effusion, Cardiac Tamponade

## Abstract

**Background:**

Pericardial window, in addition to promoting pericardial drainage, can also provide samples of the pericardium for anatomopathological examination. However, such biopsies’ contribution to clarifying the etiology of pericardial effusion has been debated.

**Objective:**

To analyze the diagnostic value of non-targeted pericardial biopsy obtained from pericardial window procedures.

**Methods:**

Data from 80 patients who had undergone parietal pericardial biopsies from 2011 to 2020 were retrospectively reviewed. Statistical significance was considered if p < 0.05.

**Results:**

Fifty patients were male (62.5%,) and 30 were female (37.5%). The median age was 52 years (interquartile range: 29 to 59) and 49 years (interquartile range: 38 to 65), respectively (p = 0.724). The suspected etiology of pericardial effusion was neoplastic in 31.3%, unclear in 25%, tuberculosis in 15%, autoimmune in 12.5%, edemagenic syndrome in 7.5%, and other miscellaneous conditions in 8.8%. The most frequent approach for pericardial drainage and biopsy was subxiphoid (74%), followed by video-assisted thoracoscopy (22%). Overall, in 78.8% of the biopsies, the histopathologic findings were compatible with nonspecific inflammation, and only 13.7% of all biopsies yielded a conclusive histopathological diagnostic. Those suffering from cancer and pericardial effusion had a higher proportion of conclusive histopathologic findings (32% had pericardial neoplastic infiltration). The hospital mortality rate was 27.5%, and 54.5% of the patients who died in the hospital had cancer. No deaths were attributed to cardiac tamponade or the drainage procedure.

**Conclusion:**

Our results showed that pericardial window is a safe procedure, but it had little value to clarify the pericardial effusion etiology and no impact on the planned therapy for the primary diagnosis besides the cardiac decompression.

**Central Illustration:** : Pericardial Windows: The Limited Diagnostic Value of Non-Targeted Pericardial Biopsy

## Introduction

Pericardial effusion is not an uncommon condition. It may be asymptomatic or show clinical signs of cardiovascular functional impairment due to cardiac tamponade. The etiology of pericardial effusion varies according to demographic characteristics and existing comorbidities. Infections, cardiac surgery, inflammatory/rheumatological, neoplastic and idiopathic are the most cited causes,^1^and many are associated with known medical conditions, such as chronic renal failure and other edematogenic syndromes.

Subxiphoid pericardiocentesis, first described by Marfan,^2^is a minimally invasive procedure that can be performed with local anesthesia at the “bedside.” In addition to decompressing the heart, it provides a fluid sample for diagnostic purposes. However, this method usually does not provide samples of pericardial tissue for anatomopathological examination.

Pericardial window, or fenestration, is a procedure performed by means of several open surgical approaches (subcostal, sternotomy, thoracotomy, thoracoscopic, subxiphoid), which, in addition to promoting pericardial drainage, can also provide samples of the pericardium for anatomopathological examination. The subxiphoid approach, known as “subxiphoid window,” is commonly used, since it does not require special instruments and equipment, preserves the pleural space and sternum, and may be performed with local anesthesia.

Despite the effectiveness of pericardial window to resolve cardiac tamponade, the contribution of the standard, non-targeted, pericardial samples obtained using such approaches to clarify the effusion etiology is questionable.^3 , 4^

Therefore, the aim of this study was to analyze the diagnostic value of non-targeted pericardial biopsy obtained from pericardial window procedures.

## Methods

All parietal pericardial specimens surgically excised at the Hospital das Clínicas of the School of Medicine of Ribeirão Preto of the University of São Paulo, Brazil, from 2011 to 2020 were reviewed. The clinical history and course, as well echocardiography and histological results were retrospectively obtained by accessing patients’ medical records.

All biopsies were obtained during pericardial window performed by the subxiphoid approach, video-assisted thoracoscopy, or thoracotomy under general anesthesia. This study was approved by the research ethics committee of the Hospital das Clínicas of the School of Medicine of Ribeirão Preto of the University of São Paulo (CAAE: 65868422.3.0000.5440).

### Statistical Analysis

The distribution of data was screened using histograms and Q-Q plots. Since the continuous variables were not normally distributed, their results are presented as medians and first and third quartiles (Q1 to Q3). The results for categorical variables are presented as proportions. Continuous and categorical variables were compared using the Mann-Whitney test and the Fisher exact test, respectively. Statistical significance was considered when p < 0.05. The analysis was performed using SPSS for Windows (IBM® SPSS®), version 25.

## Results

Data from 80 patients were reviewed. Fifty patients were male (62.5%), and 30 were female (37.5%). The median age was 52 years (interquartile range: 29 to 59) and 49 years (interquartile range: 38 to 65), respectively (p = 0.724). According to echocardiographic parameters, pericardial effusion was deemed small in 17% of the patients, moderate in 28%, and large in 55%. Concomitant pleural effusion was present in 59% of the patients, regardless of pericardial effusion volume (p = 0.394). Only 5% of all patients needed urgent pericardial drainage.

The suspected etiology of pericardial effusion, based solely on the clinical history and non-invasive diagnostic exams, was neoplastic in 31.3%, unclear in 25%, tuberculosis in 15%, autoimmune in 12.5%, edemagenic syndrome (cirrhosis, congestive heart failure, consumptive syndrome, renal failure) in 7.5%, and other miscellaneous conditions in 8.8%.

Overall, 11 patients (13.8%) had previous pericardial drainage, of which 63.6% were through subxiphoid pericardiocentesis and the others through subxiphoid window. Among patients with previous pericardial drainage, 45.5% had cancer; 18.2% had edemagenic syndrome, and the clinical diagnosis was unclear in 18.2%. Table 1 shows the clinical characteristics and symptoms of all patients.

**Table 1:** – Clinical characteristics and symptoms of patients

Left ventricle ejection fraction		0.65 (0.57-0.69)
Hemoglobin (g/dl)		10.9 (9-13)
Hematocrit (%)		32.5 (28-39)
Platelets (× 10^3^/µl)		286 (193-380)
Leucocytes (× 10^3^/mm^3^)		8 (5-12)
Reactive C-protein (mg/L)		7.3 (2-17)
Jugular venous distension		22.7%
Muffled heart sounds		16.7%
Paradoxical pulse		1.5%
Systolic blood pressure < 100 mmHg		10.8%
Beck’s triad		0.0%
Dyspnea		43.9%
Orthopnea		13.6%
Paroxysmal nocturnal dyspnea		6.1%
NYHA class	I	62.7%
	II	23.7%
	III	8.5%
	IV	5.1%
Fever		10.6%
Chest pain		13.6%
Ascites		7.5%
Pericardial friction		1.5%
Fatigue		28.8%
Pleural effusion		65.3%
Previous pericardial effusion		15.5%
Hypothyroidism		16.7%
Cancer		33.8%
Renal failure		22.5%

Continuous variables are presented as medians and quartiles (Q1 to Q3).

The most frequent approach for pericardial drainage and biopsy was subxiphoid (74%), followed by video-assisted thoracoscopy (22%) and thoracotomy (4%). Regarding the echocardiographic findings associated with cardiac tamponade,^5^overall, 34% had systolic right atrial collapse; 24% had diastolic right ventricular collapse; 46% had a decrease of 25% or more in mitral valve inflow velocity with inspiration; 41% had an increase of at least 40% in tricuspid valve inflow velocity with inspiration, and 43% did not have collapse of any chamber (right atrial or ventricle). The symptoms, signs, and echocardiographic findings according to the effusion volume determined by the transthoracic echocardiogram are shown in Table 2 .

**Table 2:** – Symptoms, signs, and echocardiographic findings according to the effusion volume determined by the transthoracic echocardiogram

Symptoms and signs	Volume							p

	Small			Moderate		Large		
		
		n	%	n	%	n	%	
Jugular venous distension		2	20.0%	2	11.1%	11	31.4%	0.252
Muffled heart sounds		1	10.0%	2	11.1%	8	22.9%	0.586
Paradoxical pulse		1	10.0%	0	0.0%	0	0.0%	0.159
Systolic blood pressure < 100 mmHg		0	0.0%	2	11.1%	5	14.7%	0.655
Beck’s triad		0	0.0%	0	0.0%	0	0.0%	-
Dyspnea		5	50.0%	6	33.3%	18	51.4%	0.418
Orthopnea		0	0.0%	4	22.2%	5	14.3%	0.334
Paroxysmal nocturnal dyspnea		1	10.0%	2	11.1%	1	2.9%	0.360
Exertion		6	60.0%	5	27.8%	8	22.9%	0.091
NYHA class	I	6	60.0%	12	70.6%	17	56.7%	0.907
	II	3	30.0%	4	23.5%	7	23.3%	
	III	1	10.0%	1	5.9%	3	10.0%	
	IV	0	0.0%	0	0.0%	3	10.0%	
Systolic RA collapse		0	0.0%	7	35.0%	17	44.7%	0.029* 0.004†
Diastolic RV collapse		1	8.3%	4	20.0%	12	31.6%	0.284
25% decrease in MV inflow velocity during inspiration		2	16.7%	8	40.0%	22	57.9%	0.019†
40% increase in TV inflow velocity during inspiratio		2	16.7%	4	20.0%	23	60.5%	0.018† 0.005‡

*p value for small versus moderate; † p value for small versus large; ‡ p value for moderate versus large; MV: mitral valve; RA: right atrium; RV: right ventricle; TV: tricuspid valve.

Only 13.7% of all pericardial biopsies yielded a conclusive histopathological diagnosis. Table 3 shows the predominant histopathologic findings for each suspected etiology of pericardial effusion according to the clinical history. Overall, in 78.8% of the biopsies, the histopathologic findings were compatible with nonspecific pericardial inflammation. Of all patients with pericardial effusion and cancer, 34.6% had pericardial infiltration by neoplastic cells; 57.7% had nonspecific inflammatory findings, and no abnormalities were found in 7.7%. The most frequent neoplasms associated with pericardial effusion were hematologic malignancies in 30.8%, lung cancer in 26.9%, and breast cancer and cervical cancer in 11.5% each. The higher proportion of biopsies with neoplastic pericardial involvement were those taken from patients with lung or breast cancer (positivity of 71.4% and 66.7%, respectively).

**Table 3:** – Predominant histopathologic findings for each suspected etiology of pericardial effusion according to clinical history

Suspected etiologies	Histopathologic finding									

	No abnormalities		Nonspecific inflammatory findings				Amyloidosis		Neoplastic infiltration	

			Chronic		Acute					

	n	%	n	%	n	%	n	%	n	%
Unclear diagnosis	2	10.0%	12	60.0%	5	25.0%	0	0.0%	1	5.0%
Neoplastic	2	8.0%	15	60.0%	0	0.0%	0	0.0%	8	32.0%
Autoimmune disease	2	20.0%	7	70.0%	1	10.0%	0	0.0%	0	0.0%
Tuberculosis	0	0.0%	10	83.3%	2	16.7%	0	0.0%	0	0.0%
Others	0	0.0%	6	85.7%	0	0.0%	1	14.3%	0	0
Edemagenic syndrome	0	0.0%	3	50.0%	2	33.3%	1	16.7%	0	0.0%

The overall hospital mortality rate was 27.5%, with a median age of 52 years (44 to 67), and 68.2% was female. No deaths were attributed to cardiac tamponade or the drainage procedure, and 54.5% of those who died in the hospital had cancer. The remaining deaths occurred in patients with multiple chronic comorbidities. Besides the diagnosis of cancer (50% mortality versus 16.7%, p = 0,003), no other clinical or echocardiographic variables were associated with hospital death ( Table 4 ).

**Table 4:** – Symptoms, signs and echocardiographic findings according to the outcome

		Survival		Hospital death		p

		n		n		
Age (years)		58	49 (35-59)	22	52 (44-67)	0.380
Female		35	60.3%	15	68.2%	0.610
systolic RA collapse		17	33.3%	7	36.8%	0.784
diastolic RV collapse		11	21.6%	6	31.6%	0.531
25% decrease in MV inflow velocity		0	0.0%	0	0.0%	-
40% increase in TV inflow		22	43.1%	10	52.6%	0.592
systolic RA collapse		19	37.3%	10	52.6%	0.283
Effusion volume	Small	8	15.4%	4	21.1%	0.872
	Moderate	15	28.8%	5	26.3%	
	Large	29	55.8%	10	52.6%	
Left ventricle ejection fraction		0.65 (60-69)		0.62 (0.55-0.70)		0.466
Jugular venous distension		10	20.4%	5	29.4%	0.508
Muffled heart sounds		8	16.3%	3	17.6%	0.900
Paradoxical pulse		1	2.0%	0	0.0%	1.0
Systolic blood pressure < 100 mmHg		5	10.4%	2	11.8%	1.0
Beck’s triad		19	38.8%	10	58.8%	0.169
Dyspnea		5	10.2%	4	23.5%	0.220
Orthopnea		2	4.1%	2	11.8%	0.271
NYHA class	I	29	64.4%	8	57.1%	0.854
	II	10	22.2%	4	28.6%	
	III	4	8.9%	1	7.1%	
	IV	2	4.4%	1	7.1%	

Continuous variables are presented as medians and quartiles (Q1 to Q3). MV: mitral valve; RA: right atrium; RV: right ventricle; TV: Tricuspid valve.

## Discussion

Our results showed that the pericardial window is a safe procedure, but non-targeted pericardial samples obtained from the pericardial window performed had little value to clarify the pericardial effusion etiology and no impact on the planned therapy for the primary diagnosis besides the cardiac decompression. Our results also showed that 43% of the patients had no echocardiographic signs of any chamber collapse (right atrium or ventricle), a finding with a 90% negative predictive value for tamponade.^5^

The usefulness of non-targeted pericardial biopsies obtained as part of therapeutic procedures, such as pericardial windows, has been questioned. Fernandes et al.^6^found that the pericardial biopsy revealed the etiology of the pericardial effusion in only 10.5% of 38 patients. In the experience of Boldes et al.,^3^fibrosis was found in 71% of all specimens collected; inflammatory findings were present in 86.2%, and the diagnostic value of pericardial biopsy in metastatic neoplasms of the pericardium had an overall sensitivity of 57.69%. The authors concluded that those biopsies did not change the primary diagnosis. Volk et al.^4^also observed that diagnosis was possible in only 23.9% with surgical drainage of pericardial effusion performed through a subxiphoid or thoracotomy approach.

Surgical drainage of pericardial effusion and pericardial biopsy performed through a subxiphoid approach usually allow access to a limited portion of the pericardium, decreasing the chance of obtaining representative samples. However, pericardioscopy seems to improve the diagnostic value of the pericardial biopsies, since it allows a broader inspection of the pericardial cavity, with visualization of suspected areas, consequently making it possible to obtain multiple targeted samples.^7 , 8^

Besides the contribution of the pericardial biopsy at the time of the pericardial window for clarifying the etiology, the ideal strategy for the drainage of pericardial effusions, whether pericardiocentesis or surgical drainage, also deserves further analysis.

**Figure 1:** – Histopathologic findings. A) Normal: Parietal layer of serous pericardium, fibrous pericardium and adjacent adipose tissue without histopathological changes; B) Neoplasm: Fibrous pericardium infiltrated by metastasis of high-grade osteosarcoma; C) Chronic inflammation: Fibrous pericardium exhibiting dense perivascular lymphoplasmacytic chronic inflammatory infiltrate; D) Acute inflammation: Pericardium exhibiting predominantly perivascular neutrophilic inflammatory infiltrate with extravasation of red blood cells.

Horr et al.^9^compared the outcomes of patients undergoing pericardiocentesis or pericardial window and concluded that both procedures are safe and effective strategies for patients with pericardial effusion. They also found that re-accumulation of the effusion was associated with the absence of a drain left in place after the procedure.^10^However, it is worth keeping in mind that percutaneous pericardial catheter drainage is feasible and safe, mainly if echo-guided, and that the patient’s clinical characteristics certainly have a great influence on the rate of effusion recurrence. Moreover, an extended time of pericardial catheter drainage has been associated with a reduced recurrence of pericardial tamponade after pericardiocentesis.^11^

Pan et al.,^12^using a nationally representative sample of 44,637 records, compared the outcomes between both drainage approaches, pericardiocentesis or open surgical drainage, in patients with non-surgically related pericardial effusions. They observed, after adjusting risk, that pericardiocentesis was associated with greater odds of mortality, cardiac complications, reintervention, and 30-day readmission for surgical drainage. Pericardiocentesis was associated with lower odds of infectious, respiratory, and bleeding complications, but higher odds of cardiac complications, compared to the open surgical approach.

Therefore, the debate over the best approach to diagnosing and managing pericardial effusion warrants further studies.

The present study has several important limitations. This is a retrospective analysis of patients’ medical records; therefore, it is subject to the biases of this type of study. Additionally, our cohort is small; we analyzed data only during the hospitalization period, and the choice of the strategy to approach the pericardial effusion was influenced by the operator experience, available hospital resources, and acuteness of the patient condition, resulting in potential bias.

Nevertheless, our study provides reliable data regarding the histopathologic findings from pericardial biopsies obtained through usual surgical approaches, mainly the subxiphoid, which support our conclusion and may be useful for the development of strategies to approach pericardial diseases.

## Conclusion

Our results showed that non-targeted pericardial biopsy performed at the time of pericardial window is safe, but it had little value to diagnose the etiology of the pericardial effusion.
